# MicroRNA-126 and epidermal growth factor-like domain 7 predict recurrence in patients with colon cancer treated with neoadjuvant chemotherapy

**DOI:** 10.20517/cdr.2019.08

**Published:** 2019-09-19

**Authors:** Torben Frøstrup Hansen, Anting Liu Carlsen, Julia Tanas Tanassi, Ole Larsen, Flemming Brandt Sørensen, Lars Henrik Jensen, Anders Jakobsen

**Affiliations:** ^1^Danish Colorectal Cancer Center South, Vejle Hospital, Institute of Regional Health Research, University of Southern Denmark, Vejle 7100, Denmark.; ^2^Department of Autoimmunology and Biomarkers, Statens Serum Institut, Copenhagen 2300, Denmark.; ^3^Department of Congenital Disorders, Statens Serum Institut, Copenhagen 2300, Denmark.; ^4^Department of Oncology, Herlev Hospital, Herlev 2730, Denmark.; ^5^University Institute of Pathology, Aarhus University Hospital, and department of Clinical Medicine, University of Aarhus, Aarhus 8200, Denmark.

**Keywords:** Chemotherapy, colon cancer, epidermal growth factor-like domain 7, microRNA-126

## Abstract

**Aim:** Neoadjuvant chemotherapy may represent a shift in the treatment of locally advanced colon cancer. The angiogenic couple has-microRNA-126 (miRNA-126) and epidermal growth factor-like domain 7 (EGFL7) are transcribed from the same gene and regulates all aspects of angiogenesis and may influence the ability of tumor cells to disseminate. The aim was to analyze the relationship between miRNA-126 and EGFL7 and disease recurrence in patients with locally advanced colon cancer treated with neoadjuvant chemotherapy.

**Methods:** This study included 71 patients from a phase II study all planned for three cycles of capecitabine and oxaliplatin before surgery. Blood was sampled at baseline and right before and after the operation. Circulating miRNA-126 was analysed by RT-qPCR and a quantitative immunoassay was used for the analyses of EGFL7.

**Results:** The rates of 5-year disease-free survival (DFS) and overall survival (OS) were 80% and 85%, respectively. The level of circulating miRNA-126 before the operation predicts recurrence, *P* = 0.035. In patients with values below and above the median the recurrence rate was 31% and 4%, respectively. Similar results applied to EGFL7. A combined estimate identified a subgroup of patients (25 of 71) with no recurrence and a 5-year DFS and OS rate of 100%, respectively.

**Conclusion:** MicroRNA-126 and EGFL7 are predictors for disease recurrence in patients with locally advanced colon cancer treated with neoadjuvant chemotherapy and may assist in selection of adjuvant chemotherapy.

## Introduction

Patients operated for high risk stage II and III colon cancer are candidates for offering adjuvant chemotherapy. Only a minor fraction of the patients benefit from this treatment modality, but nearly all suffer from the side effects, especially when treated with combination chemotherapy^[1]^. Consequently, neoadjuvant chemotherapy is currently under investigation in patients with locally advanced colon cancer as a new treatment approach. This strategy ensures early systemic treatment of minimal disease, while simultaneously providing information about tumor responsiveness^[2]^. The initial phase II experience indicates that a substantial proportion of these patients may convert to a low-risk status at the time of surgery, eliminating the need for adjuvant chemotherapy^[3]^.

Angiogenesis is a key process of tumor growth and the subsequent dissemination of tumor cells, and numerous pro- and anti-angiogenic factors are involved in its regulation^[4,5]^. We have previously focused on microRNA-126-3p (miRNA-126) and epidermal growth factor-like domain 7 (EGFL7), which are transcribed from the same gene. The cross-talk regulation between miRNA126 and EGFL7 is extremely complex and studies indicate that epigenetic modifications may regulate the expression of both parameters although other studies argue for an independent regulation^[6-9]^. This unique couple is involved in several parts of the angiogenic process including endothelial cell (EC) proliferation, migration and formation of angiogenic sprouts^[10-13]^. Overall, miRNA-126 is especially involved in the EC/blood vessel related changes while EGFL7 provides a tight connection to the extracellular matrix. A dual approach to both miRNA-126 and EGFL7 will thus encompass the entire sprouting process characteristic for the activated endothelium. A prognostic impact of miRNA-126, and to a certain degree EGFL7, has been demonstrated both in localized as well as metastatic colorectal cancer^[14-17]^, but their clinical importance in the neo-adjuvant setting is still unknown.

The aim of this study was to analyze the ability of miRNA-126 and EGFL7 to predict disease recurrence in patients with locally advanced colon cancer treated with neoadjuvant chemotherapy.

## Methods

This study follows the guidelines presented in REMARK^[18]^.

### Study population

This study is based on a phase II trial of neoadjuvant chemotherapy in patients with locally advanced colon cancer^[3]^. The present study population is identical to that of the original trial, consisting of 71 patients that completed neoadjuvant chemotherapy which was followed by surgery between August 2010 and September 2013. In brief, all patients were diagnosed with locally advanced, but resectable, colon cancer as determined by CT scans. The treatment of patients with documented wild-type *KRAS*, *BRAF*, and *PIK3CA* was supplemented with anti-EGFR treatment, panitumumab, while patients with any mutations in these genes, or unknown mutational status, were treated with neoadjuvant chemotherapy only. The selection criteria included; age ≥ 18 years, a performance status no higher than two and staged with the tumour T-category of T_3_ with extramural tumour invasion > 5 mm, or T4, based on the diagnostic CT scans, using 64-channel multidetector equipment with a section thickness of 3 mm. Inclusion and exclusion criteria and follow-up have previously been specified^[3]^. Patients were initially followed for up to three years post-operatively, according to the protocol. It was possible to extend this period to five years in relation to the present study including follow-up data on survival and histopathological verified recurrence of all the enrolled patients. All data recordings were performed as defined with good clinical practice. The study, and the present translational research, was approved by the Regional Committee on Health Research Ethics for Southern Denmark (S-20100014) and the Danish Data Protection Agency. Written informed consent was obtained, prior to inclusion, from all enrolled patients (ClinicalTrials.gov NCT01108107). Blood for the translational research was sampled at the same time with routine, treatment related blood sampling.

### Treatment

The neoadjuvant treatment was capecitabine 1000 mg/m^2^ orally two times daily on days 1-14 (28 doses) of a 21-day cycle combined with oxaliplatin 130 mg/m^2^ as a 2-h intravenous infusion on the first day of each cycle. Panitumumab was added in a dose of 9 mg/kg on the first day of each cycle, for patients with wild-type mutational tumour status. Resection of the tumour took place three weeks after the last neoadjuvant chemotherapy. Patients that fulfilled the Danish Colorectal Cancer Group criteria for adjuvant treatment (presence of lymph node metastases, a pT4 tumour, acute surgery due to obstruction of the bowel, neuronal invasion, vascular invasion, high histopathological malignancy grade, and the removal of less than 12 lymph nodes) was offered additional five cycles of the same treatment but without panitumumab. The patients that did not fulfil these criteria were not treated any further. The patients were followed for three years as previously specified^[3]^.

### Sampling

Sampling of peripheral blood was carried out before initiation of the neoadjuvant treatment (baseline), after completion of the neoadjuvant treatment, i.e. one or two days before the operation (operation), and at the first follow-up visit approximately four weeks after the operation. Sample availability varied between 75% and 87% [Supplementary Figure 1]. Venous blood (whole blood) was drawn from the antecubital area. Samples for serum (EGFL7) analyses were collected in 6 ml dry glasses, left for minimum 30 min for a clot to form, spun down for 10 min at 2500 *g*, followed by the transfer of serum to Greiner tubes (SIGMA-ALDRICH, USA) and finally frozen at -80 °C. Blood intended for plasma (miRNA-126) analyses were collected in 6 mL EDTA-containing tubes, spun down for 10 min at 2500 *g* at room temperature, and plasma was transferred and stored similar to the serum samples. The median storage time from blood sampling to analysis was 2.6 years. Samples were transported on ice from storage to analysis.

### Analysis of circulating microRNA-126

Circulating miRNA-126 (cir-miRNA-126) was analysed by staff unaware of the patient outcome and in line with previous descriptions^[19]^. Briefly, the total RNA purification kit (Norgen Biotek Corp, Ontario, Canada) was used for the purification of RNA from 100 μL of each plasma sample as described in the manufacturer’s instructions with small modifications as specified earlier. Reverse transcription was performed, using the TaqMan microRNA Reverse Transcription Kit (Applied Biosystems, Foster city, CA, USA). Reverse transcription was performed on an ABI 2720 Thermal Cycler (Applied Biosystems, Foster city, CA, USA). cDNA samples and 3 TaqMan microRNA assays (hsa-miR-126-3p, cel-miR-54 and cel-miR-238) (Applied Biosystems, Foster city, CA, USA) were applied to six 384-well plates according to instructions. All the miR-assays were performed in triplicate. The real-time PCR was performed in the ViiA7 real-time PCR system (Applied Biosystems, Foster city, CA, USA), applying a standard 384 well protocol. Data processing were performed using the Applied Biosystems’ ViiA7 real-time PCR analysis software (v.1.2.3). This approach estimates total amount of stable miRNA-126 in a given sample. However, it is not possible to determine if the miRNA originates from microvesicles or from miRNA-protein complexes.

We used a two-step normalization. First we spiked-in cel-miRNAs (cel-miRNA-54 and cel-miRNA-238) for the technical normalization, then further normalization with whole set data of miRNA-126. The Cq values were then normalized as described earlier and transformed according to the 2−ΔΔCq method^[20]^. This means that the presented estimates for cir-miRNA-126 are relative values without a dimension.

### Analysis of circulating EGFL7

We used a sandwich enzyme-linked immunosorbent assay (Cloud-Clone Corp, SEL643Hu, Houston, TX, USA) to quantify circulating EGFL7 (cir-EGFL7) in the serum samples, according to the manufacturer’s protocol. In short, 100 µL of standard or sample was added to each well of a 96-well strip plate that was pre-coated with an antibody specific to EGFL7. Then incubation for 2 h at 37 °C, aspiration, and addition of detection reagent A, incubation for an additional hour at 37 °C, aspiration, and three times washing. The described step was repeated for detection reagent B, using 30 min of incubation and five washes. Substrate solution was added and incubated for 15 minutes at 37 °C followed by the addition of a stopping solution and finally absorbance reading at 450 nm. The concentrations of EGFL7 were assessed through comparisons with the standard curve followed by multiplication with the initial dilution factor (100 fold). If samples had concentrations above the standard curve they were diluted further (and multiplied accordingly).

Samples were analysed in duplicate and we used the average for comparison with the clinical data. In-house analytical coefficients of variation on three levels were < 10% for Intra-Assay and < 12% for Inter-Assay. Protein concentrations are expressed in ng/mL.

### Statistics

We report median values followed by a 95% confidence interval. The Wilcoxon rank sum test was used comparing median values. Fisher’s Exact test was used to evaluate differences between proportions. Disease free survival (DFS) was defined as the time from the operation to the first documented tumour recurrence or death of any cause. Occurrence of other malignancies led to censoring of the DFS data in six cases. Data were censored from the day of diagnoses. Accordingly, overall survival (OS) was calculated from the date of the operation to death of any cause. Adjustment for multiple comparisons was not carried out. All statistics were performed using the NCSS statistical software (NCSS Statistical Software, Kaysville, UT 84037, USA, version 2007). *P* values < 0.05 were considered significant. All tests were two-sided.

## Results

### Patient characteristics

The patient characteristics are summarized in Table 1. At the time of data analysis, i.e., at a median follow-up of 4.0 years, disease recurrence had occurred in 14 patients.

**Table 1:** Clinical and pato-anatomical characteristics, *n* = 71

Baseline		After resection	
Characteristics	*n* (%)	Characteristics	*n* (%)
Gender		Perforation	
Male	39(55)	Yes	8(11)
Female	32(45)	No	63(89)
Age (years)		Fixation	
Mean, SD	66, 11	Yes	19(27)
Range	39 – 85	No	52(73)
> Mean	41(58)	Histology	
≤ Mean	30(42)	Adenocarcinoma	55(77)
T category		Mucinous	12(17)
T3 ETI > 5 mm	60(85)	Other^a^	4(6)
T4	11(15)	Malignancy grade	
Lymph node status		1 + 2	41(58)
N_0_	1(1)	3	19(27)
N_1-2_	70(99)	Not evaluable	11(15)
Localization		Perineural invasion	
Right	40(56)	Yes	8(11)
Left	31(44)	No	63(89)
ECOG PS		Vascular invasion	
0	36(51)	Yes	18(25)
1	35(49)	No	51(72)
Mutations		Not evaluable	2(3)
*KRAS*		Tumor budding	
wild-type	53(75)	Yes	9(13)
mutated	13(18)	No	60(85)
Unknown	5(7)	Not evaluable	2(3)
*BRAF*		MSI status	
wild-type	47(66)	MSS	50(70)
mutated	17(24)	MSI	18(25)
Unknown	7(10)	Not evaluable	3(4)
*PIK3CA*		pT category	
wild-type	52(73)	pT_0_	3(4)
mutated	10(14)	pT_1_	0 (0)
Unknown	9(13)	pT_2_	12(17)
*KRAS, BRAF, PIK3CA*		pT_3_	46(65)
All wild-type	32(45)	pT_4_	10(14)
Mutated	31(44)	Lymph nodes removed	
Unknown	8(11)	Mean	30
		Range	6-97
		pN category	
		pN_0_	47(66)
		pN_1_	15(21)
		pN_2_	9(13)
		Converted^b^	
		Yes	34(48)
		No	37(52)

ECOG PS: eastern cooperative oncology group performance status; ETI: extramural tumor invasion; MSI: microsatellite instability; MSS: microsatellite stable; N: number; SD: standard deviation. Not all sums of percentages equals 100% due to rounding of data ^a^Other includes three low differentiated adenocarcinomas and one small cell/neuroendocrine carcinoma ^b^Defines the patients where neoadjuvant chemotherapy converted them from initially high-risk (needing adjuvant chemotherapy based on initial radiology) to low-risk (not fulfilling the criteria for adjuvant chemotherapy)

### MicroRNA-126

Low-level miRNA-126 levels was related to a higher pN category (baseline, *P* = 0.036), the absence of tumor fixation, perineural invasion, microsatellite stable status, and high pT category (preoperatively, *P* < 0.050), and a higher pT category (at follow-up, *P* = 0.012, Table 2). A relationship between wild type *KRAS*, *BRAF*, and *PIK3CA* and age (*P* < 0.05) was also seen [Supplementary Table 1].

**Table 2:** Circulating microRNA-126 according to pato-anatomical characteristics

Characteristics		Baseline	*P*		before operation	*P*		Follow-up	*P*
	*N*	Median (95% CI)	value	*N*	Median (95% CI)	value	*N*	Median (95% CI)	value
Perforation									
Yes	6	0.77 (0.33-6.96)	0.459	8	1.08 (0.29-2.42)	0.389	5	1.33	0.553
No	51	1.06 (0.83-1.29)		49	0.83 (0.75-0.96)		48	1.00 (0.81-1.21)	
Fixation									
Yes	17	1.06 (0.88-1.94)	0.246	15	1.50 (0.81-2.14)	0.002	12	1.43 (0.99-2.95)	0.080
No	40	1.02 (0.72-1.27)		42	0.78 (0.65-0.87)		41	0.98 (0.77-1.15)	
Histology									
Adenocarcinoma	43	1.41 (0.80-1.35)	0.369	46	0.82 (0.73-0.94)	0.429	41	1.09 (0.90-1.33)	0.216
Mucinous	10	0.83 (0.49-2.07)		8	0.91 (0.53-2.03)		9	0.81 (0.45-1.77)	
Other^a^	4	1.02		3	1.09		3	0.73	
Malignancy grade									
1+2	31	0.88 (0.70-1.32)	0.080	36	0.79 (0.59-0.94)	0.085	31	1.01 (0.79-1.35)	0.448
3	18	1.28 (0.98-1.77)		11	1.09 (0.67-1.32)		12	0.93 (0.47-1.35)	
Not evaluable	8	0.64 (0.41-2.07)		10	0.87 (0.65-2.03)		10	1.03 (0.81-1.82)	
Perineural invasion									
Yes	8	0.93 (0.33-1.32)	0.275	7	0.64 (0.29-0.94)	0.021	4	1.47	0.449
No	49	1.06 (0.83-1.31)		50	0.87 (0.78-1.08)		49	1.00 (0.85-1.22)	
Tumor budding									
Yes	8	1.16 (0.33-1.43)	0.971	8	0.83 (0.29-1.62)	0.858	5	1.09	0.950
No	47	0.98 (0.80-1.29)		47	0.83 (0.75-0.99)		47	1.01 (0.85-1.31)	
Not evaluable	2	2.79		2	1.76		1	0.99	
MSI status									
MSI	16	1.11 (0.86-1.43)	0.495	13	1.31 (0.82-1.50)	0.044	12	0.87 (0.53-2.51)	0.733
MSS	38	1.03 (0.66-1.32)		42	0.78 (0.67-0.87)		38	1.08 (0.79-1.35)	
Not evaluable	3	0.83		2	0.91		3	1.01	
pT category^b^									
pT_0_	1	1.06		2	0.72		2	2.09	
pT_1_	0			0			0		
pT_2_	7	0.83 (0.50-2.98)		10	0.79 (0.54-1.08)		9	0.90 (0.73-1.35)	
pT_3_	42	1.08 (0.80-1.32)	0.458	37	0.94 (0.82-1.32)	0.042	37	1.09 (0.89-1.35)	0.018
pT_4_	7	0.95 (0.33-2.07)		8	0.66 (0.29-0.81)		5	0.44	
pN category^c^									
pN_0_	38	1.15 (0.95-1.35)		37	0.84 (0.67-0.99)		36	1.00 (0.85-1.16)	
pN_1_	11	0.70 (0.41-2.07)	0.036	14	0.83 (0.64-1.55)	0.587	11	1.35 (0.42-1.77)	0.924
pN_2_	8	0.64 (0.49-0.93)		6	0.86 (0.31-2.03)		6	1.09 (0.39-3.86)	
Converted									
Yes	27	1.15 (0.83-1.48)	0.103	27	0.82 (0.65-0.99)	0.955	27	0.99 (0.85-1.16)	0.852
No	30	0.89 (0.62-1.27)		30	0.84 (0.76-1.09)		26	1.22 (0.48-1.77)	

CI: confidence interval; MSI: microsatellite instability; MSS: microsatellite stable; N: number.

Confidence intervals are not calculated for cases with less than six individuals N differs according to availability of blood samples [Supplementary Figure 1]. ^a^Other includes three low differentiated adenocarcinomas and one small cell/neuroendocrine carcinoma ^b^P-value refer to the comparison pT_4_ *vs*. pT_0-3_^c^*P*-value refer to the comparison pN_0_ *vs*. pN_1-2_

**Figure 1:**
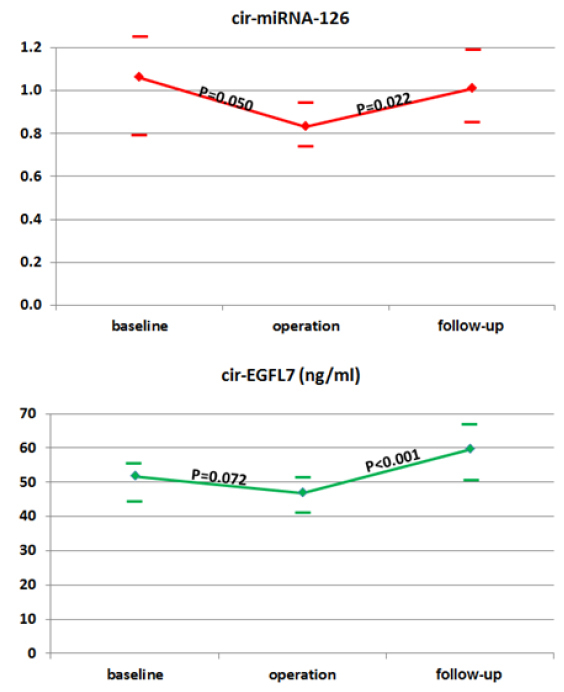
Changes in circulating microRNA-126 (cir-miRNA-126) and epidermal growth factor-like domain 7 (cir-EGFL7) during treatment. Median values are illustrated with horizontal lines marking the respective upper and lower limits of the 95% confidence intervals (CI). *P*-values refer to differences between the individual time points (baseline, operation, and follow-up) based on the Wilcoxon Signed-Rank Test for differences at the medians

Overall, the median miRNA-126 decreased significantly during neoadjuvant chemotherapy followed by an increase postoperatively [Figure 1].

### Epidermal growth factor-like domain 7

With the exception of performance status, no relationships between EGFL7 and clinical or patho-anatomical characteristics were detected [Supplementary Tables 2 and 3].

Similar to that of miRNA-126, the median EGFL7 tended to decrease during neoadjuvant chemotherapy followed by a significant increase postoperatively [Figure 1].

### Recurrence and prognosis

**Figure 2:**
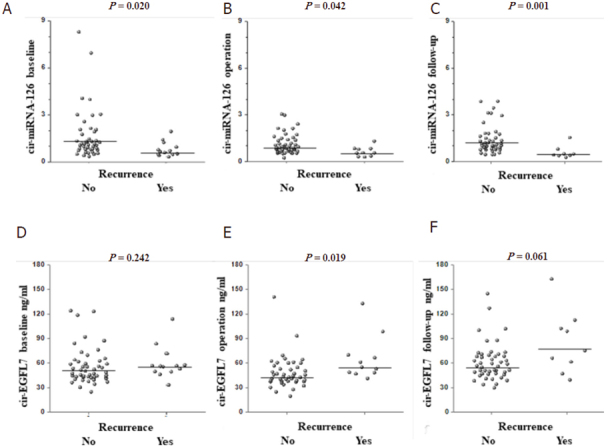
Dot plots illustrating the results from microRNA-126 (miRNA-126) A-C, and epidermal growth factor-like domain 7 (EGFL7) D-F analyses according to recurrence status, at baseline, operation, and follow-up, respectively. Horizontal bars represent medians

The distributions of miRNA-126 and EGFL7 values, according to recurrence is shown in Figure 2. Patients with disease recurrence were characterized by a significantly lower levels of miRNA-126 at all sampling points (*P* < 0.05), compared to patients without disease recurrence. Also, high levels of EGFL7 tended to be associated with disease recurrence.

In order to assess the relationship between the analyzed parameters and disease recurrence, patients were divided into two groups using the median values as cut-off. Based on these values, the recurrence rates differed significantly at the time of operation both for the miRNA-126 levels (31% *vs.* 4%, *P* = 0.035) and the EGFL7 levels (37% *vs.* 4%, *P* = 0.017), respectively [Table 3]. Using combined estimates [Table 3] led to the identification of a group at very high risk (50%) of recurrence and a no-risk (0%) group.

**Table 3:** MicroRNA-126 and EGFL7 as individual and combined estimates in relation to disease recurrence

miRNA-126 and EGFL7 as individual estimates			
Parameter	< Median	> Median	*P*-value*
miRNA-126 baseline	10/29 = 34%	3/28 = 11%	0.124
miRNA-126 before operation	9/29 = 31%	1/28 = 4%	0.035
miRNA-126 follow-up	7/27 = 26%	1/26 = 4%	0.067
EGFL7 baseline	3/31 = 10%	10/31 = 32%	0.124
EGFL7 before operation	1/28 = 4%	10/27 = 37%	0.017
EGFL7 follow-up	2/28 = 7%	7/28 = 25%	0.161
**Combined estimate of miRNA-126 and EGFL7**			
Sample**	**miRNA-126 high + EGFL7 low**	**miRNA-126 low + EGFL7 high**	*P*-value*
Baseline	1/17 = 6%	8/19 = 42%	0.064
Before operation	0/17 = 0%	8/16 = 50%	0.013
Follow-up	0/12 = 0%	4/11 = 36%	0.106

EGFL7: epidermal growth factor-like domain 7; miRNA-126: microRNA-126. * Differences between the proportions are estimated using the Fisher’s Exact test. ** Only the presumed low risk (miRNA-126 high + EGFL7 low) and high risk (miRNA-126 low + EGFL7 high) groups are depicted. Ratios and percentages refer to recurrence rates

**Figure 3:**
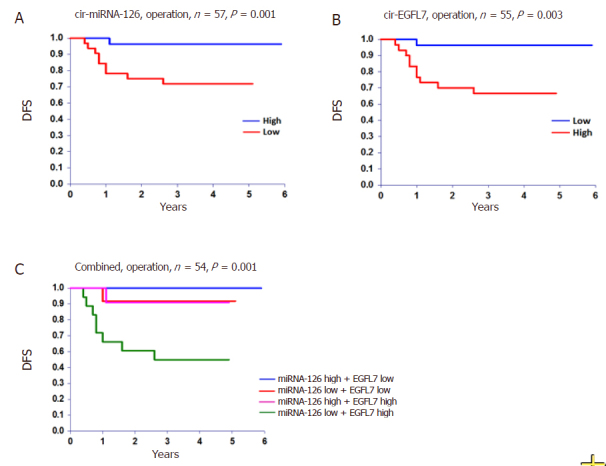
Disease free survival (DFS). The DFS at time of operation according to A, circulating microRNA-126 (cir-miRNA-126), B circulating epidermal growth factor-like domain 7 (cir-EGFL7), and C the combined estimate

For the entire cohort the rate of 5-year DFS and OS was 80% and 85%, respectively. The relationship between disease recurrence and miRNA-126 and EGFL7, at the time of operation, translated into a significant benefit as to DFS [Figure 3]. There were no significant relationships with OS, *P* = 0.149 and *P* = 0.126, respectively.

## Discussion

Neoadjuvant chemotherapy represents a new and promising treatment approach for patients with localized advanced colon cancer. While our initial phase II data are currently being validated in an ongoing phase III trial (EudraCT no: 2013-002363-26), the present translational study, with a median follow-up of four years, indicates a very promising outcome for this patient category in general. The data suggest a relationship between miRNA-126, EGFL7, and disease, which expands the possibility for treatment selection.

The present results demonstrated a significant relationship between low cir-miRNA-126 and high cir-EGFL7 levels and the clinically relevant endpoint of disease recurrence. This was especially pronounced in the “before operation” samples, which precedes the decision about offering adjuvant chemotherapy. This motivated us to generate a combined estimate focusing specifically on the presumed low- and high-risk combinations (miRNA-126 high + EGFL7 low, and miRNA-126 low + EGFL7 high). Twenty-five patients were identified as low risk, based on the combined miRNA-126/EGFL7 estimate from the “before operation” or the “follow-up” samples. None of these patients experienced disease recurrence, but 10 were still high-risk and thus received adjuvant chemotherapy. This underlines the predictive potential of these two biomarkers in the clinical setting. Although there are no directly comparable studies in the neoadjuvant setting, several preclinical studies have demonstrated relationships between low miRNA-126 expression and increased angiogenesis, tumor growth, migration, and the metastatic potential, supporting the present results^[6,21-23]^. The same pattern was seen with high cir-EGFL7. This is in agreement with our previous study analyzing EGFL7 expression in 126 patients with stage II-IV colon cancer^[17]^. It showed a significantly higher EGFL7 expression in the primary tumor of recurred patients than of those who remained recurrence free after the operation. The correlation with prognosis is well in line with previous studies in locally advanced cancer as concerns miRNA-126^[16,24]^, and with disseminated disease with respect to EGFL7^[15]^. A meaningful multiple Cox Regression analysis was not feasible with only 14 events in the DFS analyses.

The levels of miRNA-126 and EGFL7 seem to decline during treatment and rise again after the operation. This may be explained by a decreased number of immature blood vessels during treatment, which is particularly pronounced in the malignant tumor. The general increase seen in the postoperative samples are most likely also influenced by the postoperative stress response, but it is interesting that the relationship with recurrence status is also recapitulated at this time point.

An opportunity to analyze miRNA-126 expression in the diagnostic biopsies appeared after the generation of these originally planned blood-based results. This was performed by *in situ* hybridization and image guided analyses as previously described^[25,26]^ and the results suggest a similar distribution with a lower expression of miRNA-126 in the patients who eventually experienced disease recurrence supporting the presented results [Supplementary Figure 2].

Post-transcriptional gene regulation is one of the main functions related to miRNAs that often have multiple targets. This is also true for miRNA-126 and the regulation of several of these mRNA targets may all impact on the risk of disease recurrence from local colon cancer. Some of the more well described targets are *SPRED1*, *p85β*, and *PI3KR2* governing vascular integrity^[10,27]^, VEGF-A regulating AKT-pathway signaling^[28]^, CXCR4 and IRS-1 involved in CRC cell proliferation and migration^[29,30]^, and KRAS impacting on the viability of the mutated tumor cells^[31]^. Recent reviews have also highlighted the clinical potential of miRNA-126^[32-34]^.

The limitations of the current study are associated with the general caveats related to interpreting circulating biomarker data. The analysis of miRNAs is influenced by several technical aspects such as choice of blood fraction, sampling, handling, processing, normalization procedures, and the possible contamination from platelets during processing. Our results being influenced by one or more of these steps cannot be excluded, but the consistency of the presented results is remarkable. The blood sampling at baseline, surgery, and follow-up represents three distinct time points with weeks and months between them. The same significant relationship between disease recurrence and miRNA-126 is demonstrated at all three time points. This would probably not be the case, if data were strongly influenced by pre-analytical and analytical factors. Multiple comparisons, which may be inherent to this hypothesis generating study, could influence some of the correlations with the clinical and patho-anatomic characteristics. Also, blood samples from all patients at all sampling points would have been preferable, but an availability of 75%-87% is acceptable. The study population, comprising all patients enrolled in a well-described clinical phase II trial, is a strength of the study.

Our initial experience with neoadjuvant chemotherapy is that half of the patients converted from high to low-risk status during the three cycles of preoperative chemotherapy actually do surprisingly well (4-year OS rate of 90%). They are spared a substantial amount of adjuvant chemotherapy and the adverse events associated with this treatment. The identification of prognostic/predictive biomarkers in this clinical situation is of importance, as many of the unconverted patients also remain recurrence free, and thus could be spared from the adjuvant chemotherapy if identified. The analyses of miRNA-126 and EGFL7, and especially the combined estimate, may procure such information. It is well known, however, that the cross-talk regulation between miRNA-126 and EGFL7 is extremely complex and the exact interpretation of these data in a neoadjuvant setting is consequently scheduled for further validation in an ongoing phase III trial.

In conclusion, miRNA-126 and EGFL7 are predictive of disease recurrence in patients with locally advanced colon cancer treated with neoadjuvant chemotherapy and may be instrumental in the identification of patients to be spared of adjuvant chemotherapy.

## Declarations

### Availability of data and material

The datasets supporting the conclusions of this article are included within the article (and its additional files).

### Copyright

© The Author(s) 2022.

## References

[1] Andre T, Boni C, Navarro M, Tabernero J, Hickish T (2009). Improved overall survival with oxaliplatin, fluorouracil, and leucovorin as adjuvant treatment in stage II or III colon cancer in the MOSAIC trial.. J Clin Oncol.

[2] Foxtrot Collaborative Group (2012). Feasibility of preoperative chemotherapy for locally advanced, operable colon cancer: the pilot phase of a randomised controlled trial.. Lancet Oncol.

[3] Jakobsen A, Andersen F, Fischer A, Jensen LH, Jorgensen JC (2015). Neoadjuvant chemotherapy in locally advanced colon cancer. A phase II trial.. Acta Oncol.

[4] Hanahan D, Weinberg RA (2011). Hallmarks of cancer: the next generation.. Cell.

[5] Carmeliet P, Jain RK (2011). Principles and mechanisms of vessel normalization for cancer and other angiogenic diseases.. Nat Rev Drug Discov.

[6] Sun Y, Bai Y, Zhang F, Wang Y, Guo Y (2010). miR-126 inhibits non-small cell lung cancer cells proliferation by targeting EGFL7.. Biochem Biophys Res Commun.

[7] Saito Y, Friedman JM, Chihara Y, Egger G, Chuang JC (2009). Epigenetic therapy upregulates the tumor suppressor microRNA-126 and its host gene EGFL7 in human cancer cells.. Biochem Biophys Res Commun.

[8] Hu MH, Ma CY, Wang XM, Ye CD, Zhang GX (2016). MicroRNA-126 inhibits tumor proliferation and angiogenesis of hepatocellular carcinoma by down-regulating EGFL7 expression.. Oncotarget.

[9] Gong C, Fang J, Li G, Liu HH, Liu ZS (2017). Effects of microRNA-126 on cell proliferation, apoptosis and tumor angiogenesis via the down-regulating ERK signaling pathway by targeting EGFL7 in hepatocellular carcinoma.. Oncotarget.

[10] Fish JE, Santoro MM, Morton SU, Yu S, Yeh RF (2008). miR-126 regulates angiogenic signaling and vascular integrity.. Dev Cell.

[11] Wang S, Aurora AB, Johnson BA, Qi X, McAnally J (2008). The endothelial-specific microRNA miR-126 governs vascular integrity and angiogenesis.. Dev Cell.

[12] Parker LH, Schmidt M, Jin SW, Gray AM, Beis D (2004). The endothelial-cell-derived secreted factor Egfl7 regulates vascular tube formation.. Nature.

[13] Nikolic I, Stankovic ND, Bicker F, Meister J, Braun H (2013). EGFL7 ligates alphavbeta3 integrin to enhance vessel formation.. Blood.

[14] Hansen TF, Soerensen FB, Lindebjerg J, Jakobsen A (2012). The predictive value of microRNA-126 in relation to first line treatment with capecitabine and oxaliplatin in patients with metastatic colorectal cancer.. BMC Cancer.

[15] Hansen TF, Nielsen BS, Sorensen FB, Johnsson A, Jakobsen A (2014). Epidermal growth factor-like domain 7 predicts response to first-line chemotherapy and bevacizumab in patients with metastatic colorectal cancer.. Mol Cancer Ther.

[16] Hansen TF, Kjaer-Frifeldt S, Morgenthaler S, Blondal T, Lindebjerg J (2014). The prognostic value of microRNA-126 and microvessel density in patients with stage II colon cancer: results from a population cohort.. J Transl Med.

[17] Hansen TF, Nielsen BS, Jakobsen A, Sorensen FB (2015). Intra-tumoural vessel area estimated by expression of epidermal growth factor-like domain 7 and microRNA-126 in primary tumours and metastases of patients with colorectal cancer: a descriptive study.. J Transl Med.

[18] McShane LM, Altman DG, Sauerbrei W, Taube SE, Gion M (2005). REporting recommendations for tumour MARKer prognostic studies (REMARK).. Br J Cancer.

[19] Hansen TF, Carlsen AL, Heegaard NH, Sorensen FB, Jakobsen A (2015). Changes in circulating microRNA-126 during treatment with chemotherapy and bevacizumab predicts treatment response in patients with metastatic colorectal cancer.. Br J Cancer.

[20] Schmittgen TD, Livak KJ (2008). Analyzing real-time PCR data by the comparative C(T) method.. Nat Protoc.

[21] Li XM, Wang AM, Zhang J, Yi H (2011). Down-regulation of miR-126 expression in colorectal cancer and its clinical significance.. Med Oncol.

[22] Li N, Tang A, Huang S, Li Z, Li X (2013). MiR-126 suppresses colon cancer cell proliferation and invasion via inhibiting RhoA/ROCK signaling pathway.. Mol Cell Biochem.

[23] Ebrahimi F, Gopalan V, Wahab R, Lu CT, Smith RA (2015). Deregulation of miR-126 expression in colorectal cancer pathogenesis and its clinical significance.. Exp Cell Res.

[24] Liu Y, Zhou Y, Feng X, Yang P, Yang J (2014). Low expression of microRNA-126 is associated with poor prognosis in colorectal cancer.. Genes Chromosomes Cancer.

[25] Jorgensen S, Baker A, Moller S, Nielsen BS (2010). Robust one-day in situ hybridization protocol for detection of microRNAs in paraffin samples using LNA probes.. Methods.

[26] Nielsen BS, Jorgensen S, Fog JU, Solkilde R, Christensen IJ (2011). High levels of microRNA-21 in the stroma of colorectal cancers predict short disease-free survival in stage II colon cancer patietns.. Clin Exp Metastasis.

[27] Guo C, Sah JF, Beard L, Wilson JK, Markowitz SD (2008). The noncoding RNA, miR- 126, suppresses the growth of neoplastic cells by targeting phosphatidylinositol 3-kinase signaling and is frequently lost in colon cancers.. Genes Chromosomes Cancer.

[28] Zhu N, Zhang D, Xie H, Zhou Z, Chen H (2011). Endothelial-specific intron-derived miR-126 is down-regulated in human breast cancer and targets both VEGF-A and PIK3R2.. Mol Cell Biochem.

[29] Liu Y, Zhou Y, Feng X, An P, Quan X (2014). MicroRNA-126 functions as a tumor suppressor in colorectal cancer cells by targeting CXCR4 via the AKT and ERK1/2 signalling pathways.. Int J Oncol.

[30] Zhou Y, Feng X, Liu YL, Ye SC, Wang H (2013). Down-regulation of miR-126 is associated with colorectal cancer cells proliferation, migration and invasion by targeting IRS-1 via the AKT and ERK1/2 signalling pathways.. PLoS One.

[31] Hara T, Jones MF, Subramanian M, Li XL, Ou O (2014). Selective targeting of KRAS-mutant cells by miR-126 through repression of multiple genes essential for the survival of KRAS-mutant cells.. Oncotarget.

[32] Dong Y, Fu C, Guan H, Zhang Z, Zhou T (2016). Prognostic significance of miR-126 in various cancers: a meta-analysis.. Onco Targets Ther.

[33] Yan J, Ma S, Zhang Y, Yin C, Zhou X (2016). Potential role of mircroRNA-126 in the diagnosis of cancers. A systematic review and meta-analysis.. Medicine.

[34] Santulli G (2016). MicroRNAs and Endothelial (Dys) Function.. J Cell Physiol.

