# Supplementary material for: MicroRNA-126 and epidermal growth factor-like domain 7 predict recurrence in patients with colon cancer treated with neoadjuvant chemotherapy
Source: Cancer Drug Resist. 2019 Sep 19;2(3):885–96. doi: 10.20517/cdr.2019.08 (PMC8992522; doi:10.20517/cdr.2019.08)
Supplement: Supplementary file 1 [file cdr-2-885-SupplementaryMaterials.pdf]

Supplementary

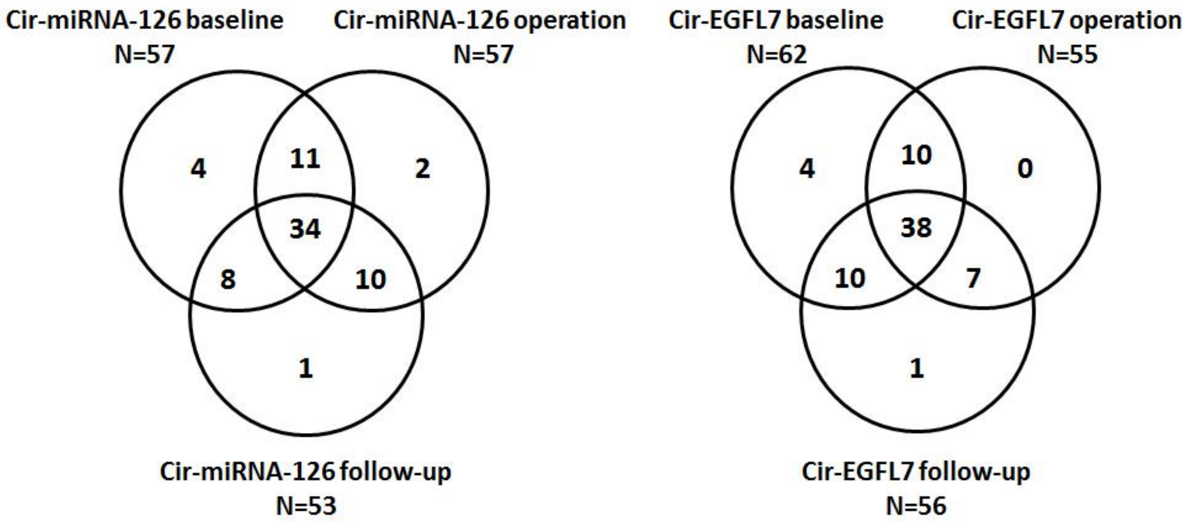

Supplementary Figure 1. Blood sample distributions. Patient adherence to the blood sampling varied throughout the trial period and explains why N is not 71 at all the sampling time points

Cir-miRNA-126: Circulating microRNA-126; cir-EGFL7: Circulating epidermal growth factor like domain 7.

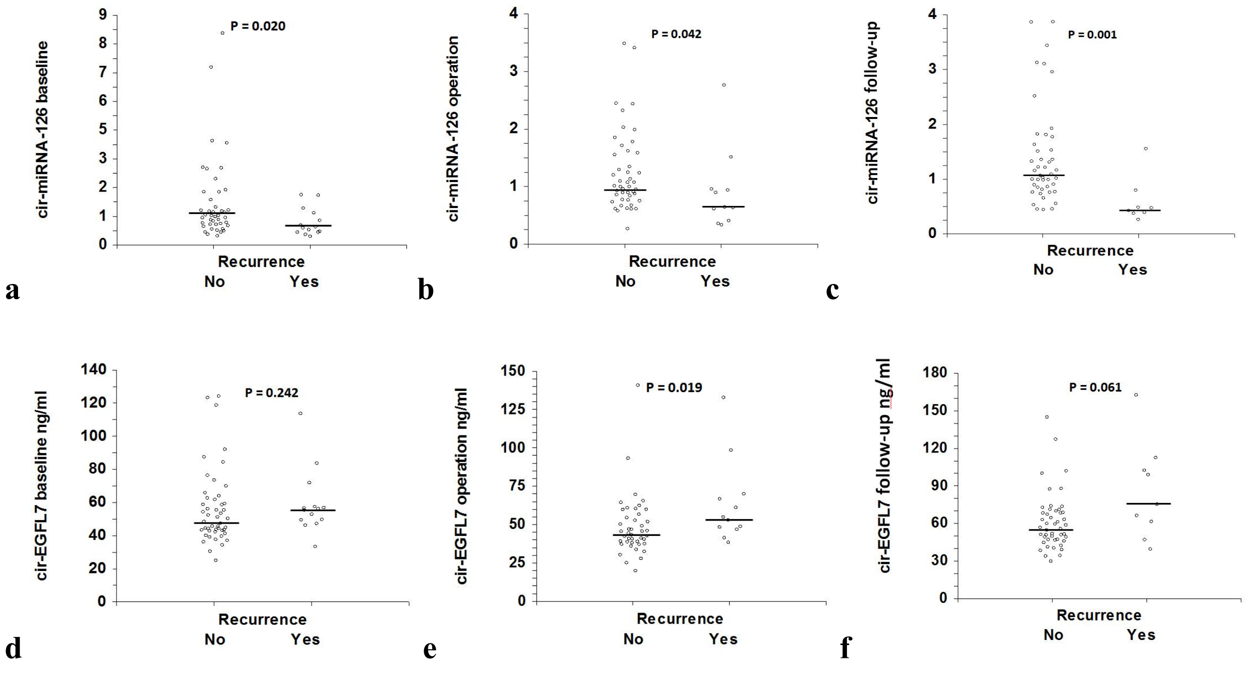

Supplementary Figure 2. Dot plots illustrating the results from microRNA-126 (miRNA-126) **a-c**, and epidermal growth factor-like domain 7 (EGFL7) **d-f** analyses according to recurrence status, at baseline, operation, and follow-up, respectively

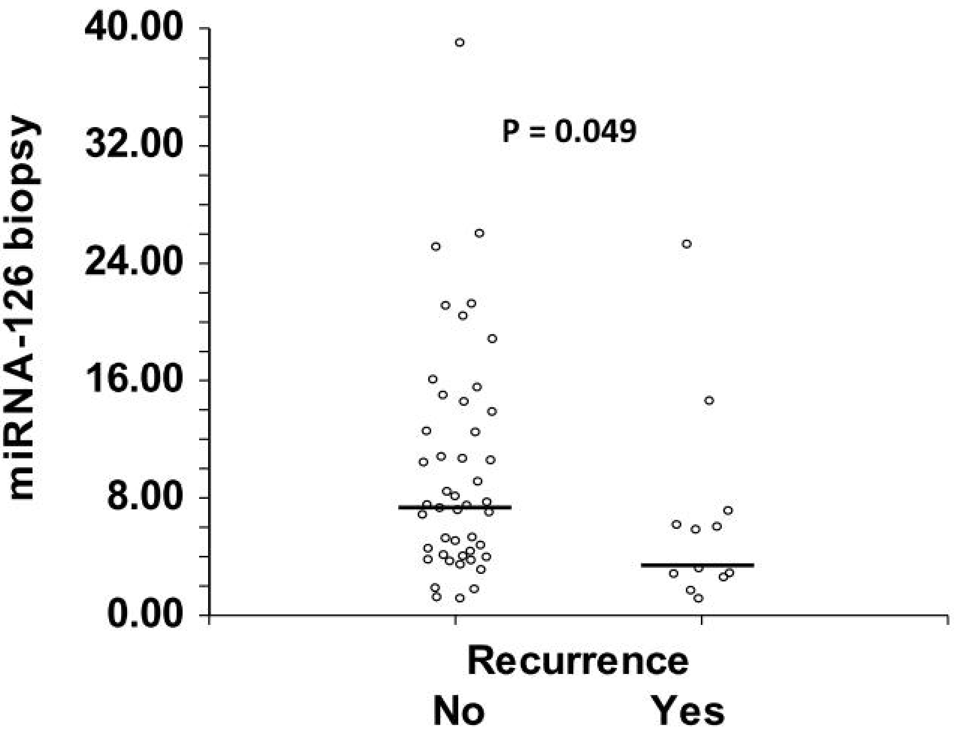

Supplementary Figure 3. Dot plot illustrating the expression levels of microRNA-126 (miRNA-126) in the diagnostic biopsies according to recurrence status

Supplementary Table 1. MicroRNA-126 according to clinical characteristics

Characteristics	N	*Baseline* Median (95% CI)	*p* value	N	*before operation* Median (95% CI)	*p* value	N	*Follow-up* Median (95% CI)	*p* value
**Gender**									
Male	30	0.97 (0.70-1.35)	0.570	29	0.78 (0.55-1.08)	0.228	27	0.90 (0.65-1.33)	0.247
Female	27	1.10 (0.77-1.32)		28	0.90 (0.78-1.09)		26	1.08 (0.89-1.53)	
**Age** (years)									
> Mean	29	1.06 (0.59-1.77)	0.756	24	0.73 (0.55-0.96)	0.106	24	0.80 (0.65-1.06)	**0.027**
≤ Mean	28	1.04 (0.59-1.77)		33	0.87 (0.78-1.32)		29	1.16 (0.99-1.51)	
**T category**									
T3 ETI > 5 mm	47	0.99 (0.80-1.25)	0.476	50	0.84 (0.76-0.99)	0.458	46	1.01 (0.79-1.22)	0.563
T4	10	1.39 (0.49-2.98)		7	0.82 (0.23-1.77)		7	1.15 (0.55-2.95)	
**Localization**									
Right	33	1.06 (0.80-1.32)	0.728	31	0.84 (0.65-1.17)	0.689	31	0.98 (0.76-1.31)	0.206
Left	24	1.04 (0.62-1.35)		26	0.81 (0.67-1.05)		22	1.09 (0.81-1.55)	
**ECOG PS**									
0	30	1.12 (0.77-1.48)	1.000	29	0.84 (0.73-1.31)	0.844	26	1.01 (0.85-1.22)	0.936
1	17	1.06 (0.75-1.77)		21	0.88 (0.53-1.17)		19	1.21 (0.73-1.35)	
**Mutations**									
KRAS									
wild-type	41	1.10 (0.77-1.32)	0.702	43	0.83 (0.73-0.99)	0.293	39	1.01 (0.81-1.22)	0.275
mutated	12	0.82 (0.59-1.94)		11	0.88 (0.56-1.62)		10	0.85 (0.42-1.51)	
Unknown	4	0.96		3	0.67		4	1.30	
BRAF									
wild-type	36	1.01 (0.70-1.35)	0.885	41	0.81 (0.67-1.05)	0.232	37	1.09 (0.90-1.35)	0.164
mutated	15	1.06 (0.77-1.43)		12	0.97 (0.79-1.38)		10	0.79 (0.47-2.51)	
Unknown	6	0.96 (0.55-3.99)		4	0.77		6	1.11 (0.45-3.10)	
PIK3CA									
wild-type	39	0.99 (0.70-1.27)	0.157	44	0.80 (0.73-1.08)	0.538	37	1.03 (0.86-1.35)	0.191
mutated	10	1.32 (0.72-4.08)		7	0.94 (0.53-1.74)		8	0.92 (0.26-1.22)	
Unknown	8	0.96 (0.55-1.96)		6	0.85 (0.65-1.32)		8	1.11 (0.37-1.55)	
KRAS, BRAF, PIK3CA									
All wild-type	21	0.93 (0.62-1.35)	0.596	27	0.75 (0.55-0.84)	**0.024**	24	1.12 (0.79-1.63)	0.138
Mutated	29	1.06 (0.77-1.36)		25	0.96 (0.82-1.38)		22	0.88 (0.65-1.22)	
Unknown	7	1.06 (0.55-3.99)		5	0.87		7	1.15 (0.45-3.10)	

CI: Confidence Interval; ECOG PS: Eastern Cooperative Oncology Group performance status; ETI: Extramural tumor invasion; N: Number

Median values for patients with unknown mutational status are presented but are not included in the analyses.

Confidence intervals are not calculated for cases with less than six individuals

Supplementary Table 2. EGFL7 according to clinical characteristics (ng/ml)

Characteristics	N	*Baseline* Median (95% CI)	*p* value	N	*before operation* Median (95% CI)	*p* value	N	*Follow-up* Median (95% CI)	*p* value
**Gender**									
Male	34	51.5 (46.1-56.5)	0.475	28	48.5 (41.3-59.9)	0.170	29	64.5 (51.0-71.0)	0.110
Female	28	51.6 (42.2-58.6)		27	45.6 (38.7-51.8)		27	54.6 (45.8-61.1)	
**Age** (years)									
> Mean	31	51.1 (43.0-59.1)	0.833	23	47.0 (39.1-61.1)	0.556	26	60.5 (49.0-68.6)	0.954
≤ Mean	31	52.8 (45.6-56.1)		32	45.6 (40.6-51.8)		30	59.0 (49.7-69.8)	
**T category**									
T3 ETI > 5 mm	52	51.7 (45.6-56.1)	0.871	48	46.9 (42.6-52.7)	0.067	49	61.1 (51.6-68.2)	0.511
T4	10	52.2 (36.1-87.4)		7	37.1 (25.0-62.4		7	50.8 (29.7-87.8)	
**Localization**									
Right	35	51.1 (44.4-56.5)	0.701	29	49.0 (38.0-59.9)	0.893	31	55.8 (49.0-68.2)	0.767
Left	27	53.3 (44.3-58.7)		26	46.3 (41.1-51.8)		25	61.4 (51.3-69.8)	
**ECOG PS**									
0	32	49.0 (43.5-53.3)	0.079	29	41.3 (38.3-46.7)	**0.032**	30	57.7 (49.0-64.5)	0.314
1	20	56.3 (44.8-69.8)		19	50.3 (42.6-61.1)		17	67.2 (51.6-71.0)	
**Mutations**									
KRAS									
wild-type	46	50.7 (44.1-56.1)	0.916	41	48.3 (42.4-54.5)	0.401	43	61.4 (50.8-68.2)	0.623
mutated	12	47.9 (44.8-63.9)		11	41.3 (37.0-59.9)		9	51.0 (46.5-100.0)	
Unknown	4	71.5		3	44.4		4	64.6	
BRAF									
wild-type	40	52.0 (46.1-56.7)	0.144	39	46.8 (41.1-56.6)	0.698	37	56.7 (49.0-68.6)	0.740
mutated	16	47.0 (37.6-56.1)		12	47.8 (35.9-54.8)		13	61.1 (49.0-75.2)	
Unknown	6	56.0 (39.0-87.4)		4	40.9		6	59.6 (47.0-127.1)	
PIK3CA									
wild-type	44	49.5 (44.4-56.1)	0.664	42	47.8 (41.3-56.6)	0.368	40	60.0 (49.0-68.2)	0.729
mutated	10	53.8 (44.1-59.1)		7	45.6 (35.9-54.8)		8	53.3 (42.2-72.6)	
Unknown	8	56.0 (39.0-84.3)		6	45.2 (33.7-66.7)		8	64.8 (47.0-127.1)	
KRAS, BRAF, PIK3CA									
All wild-type	25	54.2 (43.4-62.6)	0.472	25	52.7 (42.4-60.9)	0.174	25	62.8 (47.2-71.0)	0.865
Mutated	30	50.4 (44.8-56.0)		25	45.6 (39.1-51.8)		24	55.2 (49.0-66.2)	
Unknown	7	53.3 (39.0-87.4)		5	44.4		7	59.8 (47.0-162.5)	

CI: Confidence Interval; ECOG PS: Eastern Cooperative Oncology Group performance status; ETI: Extramural tumor invasion; N: Number

Median values for patients with unknown mutational status are presented but are not included in the analyses.

Confidence intervals are not calculated for cases with less than six individuals

Supplementary Table 3. EGFL7 according to pato-anatomical characteristics (ng/ml)

Characteristics	N	*Baseline* Median (95% CI)	*p* value	N	*before operation* Median (95% CI)	*p* value	N	*follow-up* Median (95% CI)	*p* value
**Perforation**									
Yes	6	54.1 (43.4-61.7)	0.840	8	47.7 (38.0-60.0)	0.858	6	67.2 (40.3-101.8)	0.289
No	56	50.7 (45.5-56.5)		47	45.9 (41.1-52.8)		50	57.7 (50.7-64.5)	
**Fixation**									
Yes	18	54.1 (43.0-58.6)	0.593	15	42.4 (38.9-59.9)	0.484	12	70.4 (59.3-87.8)	0.076
No	44	50.7 (45.6-56.7)		40	47.7 (42.6-54.5)		44	55.2 (49.7-62.8)	
**Histology**									
Adenocarcinoma	47	54.2 (45.6-56.7)	0.565	45	46.8 (42.4-54.5)	0.284	42	60.2 (49.7-68.2)	0.437
Mucinous	11	48.4 (41.1-61.7)		7	41.3 (19.8-93.2)		10	53.1 (47.0-64.5)	
Other[a]	4	43.3		3	43.2		3	78.0	
**Malignancy grade**									
1+2	34	55.7 (45.5-58.7)	0.064	35	48.6 (42.6-56.6)	0.207	34	58.0 (46.9-67.2)	0.206
3	19	45.6 (41.1-52.2)		11	40.8 (25.0-60.9)		12	63.7 (51.3-87.3)	
Not evaluable	9	52.8 (40.0-69.8)		9	45.6 (37.1-51.8)		10	56.6 (47.0-127.1)	
**Perineural invasion**									
Yes	8	54.7 (33.3-76.3)	0.622	6	46.5 (41.1-132.9)	0.395	5	66.2	0.954
No	54	49.9 (45.4-56)		49	46.7 (40.8-52.7)		51	59.3 (51.0-67.2)	
**Tumor budding**									
Yes	9	53.3 (45.5-57.4)	0.983	8	51.9 (40.6-66.7)	0.139	7	69.8 (51.6-102.3)	0.050
No	51	51.1 (44.8-56.5)		45	45.9 (40.4-52.7)		48	56.3 (49.0-63.0)	
Not evaluable	2	49.2		2	43.2		1	72.6	
**MSI status**									
MSI	17	48.4 (41.1-59.1)	0.477	13	43.2 (32.3-60.4)	0.306	12	57.9 (47.0-75.2)	0.823
MSS	42	52.5 (46.1-56.5)		40	47.6 (42.6-52.8)		41	61.4 (51.3-68.2)	
Not evaluable	3	51.1		2	39.1		3	45.8	
**pT category[b]**									
pT_0	1	84.3		2	44.6		2	92.9	
pT_1	0			0			0		
pT_2	9	51.1 (44.4-73.4)	0.384	9	42.6 (38.7-64.4)	0.481	9	51.0 (38.3-68.6)	0.361
pT_3	44	49.5 (45.5-57.4)		36	46.3 (40.6-52.7)		39	59.3 (49.7-68.2)	
pT_4	8	54.1 (24.9-56.5)		8	47.7 (38.9-61.1)		6	64.6 (41.0-100.0)	
**pN category[c]**									
pN_0	41	51.1 (44.4-55.3)		36	45.0 (38.7-50.3)		38	59.2 (50.8-68.2)	
pN_1	13	47.3 (42.8-71.8)	0.582	14	50.9 (40.8-66.7)	0.072	12	49.3 (40.3-102.3)	0.833
pN_2	8	57.6 (33.3-69.8)		5	46.8		6	66.2 (46.5-112.4)	
**Converted**									
Yes	29	47.1 (43.5-55.3)	0.265	26	43.4 (38.0-51.8)	0.056	29	54.6 (49.0-67.2)	0.367
No	33	55.3 (47.3-58.7)		29	48.6 (42.6-60.4)		27	62.8 (46.9-71.0)	

CI: Confidence Interval; MSI: Microsatellite instability; MSS: Microsatellite stable; N: Number
Confidence intervals are not calculated for cases with less than six individuals
[a]Other includes three low differentiated adenocarcinomas and one small cell/neuroendocrine carcinoma
[b]P-value refer to the comparison pT_4 *versus* pT_{0-3}
[c]P-value refer to the comparison pN_0 *versus* pN_{1-2}
